# Mortality rate of patients with cystic fibrosis on the waiting list and within one year after lung transplantation: a survey of Italian CF centers

**DOI:** 10.1186/s13052-018-0512-y

**Published:** 2018-06-28

**Authors:** Beatrice Borchi, Marisol Barao Ocampo, Giuseppe Cimino, Giovanna Pizzamiglio, Silvia Bresci, Cesare Braggion, the Italian Cystic Fibrosis Lung Transplantation Group, Barbara Messore, Carlo Albera, Elisabetta Bignamini, Luca Zito, Maria Pappalettera, Carla Colombo, Valeria Daccò, Laura Minicucci, Federico Cresta, Sonia Volpi, Fiorella Battistini, Benedetta Mannozzi, Giovanna Pisi, Francesco Longo, Annalisa Cavallo, Anna Silvia Neri, Manuel Murciano, Vincenzina Lucidi, Enza Montemitro, Valeria Raia, Antonella Tosco, Vincenzo Carnovale, Paola Iacotucci, Antonio Manca, Giuseppina Leonetti, Giuseppe Magazzù, Maria Cristina Lucanto, Mirella Collura, Francesca Ficili

**Affiliations:** 1 Infectious Diseases Unit, Azienda Ospedaliero-Universitaria Careggi, Largo Brambilla 3, 50134 Florence, Italy; 2 Cystic Fibrosis Center, Azienda Ospedaliero-Universitaria di Verona, P.le Stefani 1, 37126 Verona, Italy; 3 Cystic Fibrosis Center – Adult Unit, Department of Medicine and Infectious Diseases, University Umberto I, Viale Regina Elena 324, 00161 Rome, Italy; 4 Cystic Fibrosis Center – Adult Unit, Fondazione IRCCS Ca’ Granda, Ospedale Maggiore Policlinico, Via F. Sforza 35, 20122 Milan, Italy; 5 Cystic Fibrosis Center, Meyer Children’s Hospital, Viale Pieraccini 24, 50139 Florence, Italy

## Abstract

**Background:**

Cystic Fibrosis (CF) Centers are involved in the decisions regarding the eligibility of CF patients with end-stage lung disease and timing for inclusion on waiting lists (WL) for lung transplantation (LT). There are currently no data on the mortality rates of Italian CF patients on WL and during the first year after LT and we aimed to assess these outcomes by surveying the CF Centers.

**Methods:**

A survey was sent to Italian CF Centers which were requested to report the age at which all CF subjects included on the WL between 2010 and 2014 were included on the list, admitted to either standard or urgent LT, or had died either while on the WL or within the first 3 and 12 months after LT. All outcomes were recorded by December 31, 2015.

**Results:**

Two hundred fifty-nine CF subjects were included on the WL during the 5-year study period. The mortality rate during the WL was 19.3% and was not associated with sex, age at inclusion on the WL or standard or urgent access to LT. 159 (61.4%) subjects underwent LT, 46 (28.9%) with urgent procedure. Deaths within the first 3 and 12 months after LT were significantly more prevalent in individuals who underwent urgent LT compared to those with standard LT (*p* < 0.01).

**Conclusions:**

The mortality of Italian CF patients, included in our survey, was about twice that reported by the National Transplant Center for all LT indications, including CF, during the same time period and despite the introduction of urgent LT. The latter was associated with an unfavorable early outcome compared to standard LT.

## Background

Lung transplantation (LT) is offered to subjects with cystic fibrosis (CF) with end-stage lung disease as a potential life-extending and life-improving treatment [1, 2]. Different predictive models for mortality have been proposed but the optimal timing for listing of CF individuals for LT is a complex task for the CF specialist [3–5].

In addition to measures which attempt to deal with the shortage of suitable lung donors, different organ allocation policies, based on medical urgency, have been proposed to avoid long waiting times and death either while on the waiting list (WL) or during the first year after LT. The lung allocation score (LAS) was implemented to prioritize patients in the United States [6]. Dedicated emergency LT procedures have been proposed as strategies in France and Italy for patients in extremely urgent medical conditions, such as those requiring mechanical ventilation (MV) or extracorporeal membrane oxygenation (ECMO) [7–10]. The urgent LT procedure is proposed only for patients already wait-listed in Italy [9, 10]. In France the “high emergency waiting list” is proposed for patients with an abrupt worsening of their respiratory function, requiring invasive mechanical ventilation (MV) and/or ECMO or who are at risk of imminent invasive MV or of having severe pulmonary hypertension [7, 8]. The inclusion on the regular waiting list was not an inclusion criterion in France, as recorded for 51.5% of patients in a recent nation-based study [8].

Italian CF Centers are involved in LT programs, by determining the timing for inclusion on the WL and by caring for patients while they await surgery. Listing criteria were the same as those suggested in the international literature [1, 2].

Due to the lack of data on the mortality rate of Italian CF subjects on the WL and during the first year after LT, we surveyed these outcomes, as recorded by Italian CF Centers.

## Methods

This retrospective cross-sectional survey included 22 Italian CF Centers. All CF subjects were referred to CF Centers to assess the timing for their inclusion on the WL and optimize their medical treatment during their wait. They were then referred to the Lung Transplant Centers, which established their eligibility for LT and inclusion on the WL. According to the Italian Cystic Fibrosis Register (ICFR) Report 2010, our survey addressed 4159 children and adult CF patients in 2010 [11].

CF Centers were asked to record all WL subjects between 2010 and 2014. The survey covered the following demographic and outcome data: year of inclusion on the WL, sex, age at inclusion on the WL, age at time of standard or urgent LT, age at time of death occurring either while on the WL or within 3 and 12 months after transplant. The outcomes were considered as of December 31, 2015.

CF Centers were requested to review systematically the medical records of all subjects included on the WL to avoid missing data. According to our aims, we chose to collect data only on mortality before and during the first year after LT.

Eleven Lung Transplant Centers were active in Italy in the period 2010–2014 [12]. In October 2010, under the supervision of the National Transplant Center, all Lung Transplant Centers created and agreed on a common protocol to identify patients requiring lung transplant priority [9, 10]. The Italian Urgent Lung Transplant program (IULTp) is reserved for patients younger than 50 years, who moved from the standard lung transplant program to urgent LT, if they required mechanical ventilation and/or extracorporeal lung support. Exclusion criteria are sepsis, multiorgan failure, hemorrhagic shock, neurological damage and invasive respiratory support lasting more than 14 days. Since 2010 both the standard LT program and the IULTp have been active in Italy. Priority of the IULTp can last 3 weeks. All available lungs in the country must be considered for urgent transplant first.

The yearly number of LTs, deceased patients on waiting lists and patients still on the WL for all LT indications, including patients with CF, are recorded in the Reports of the National Transplant Center [12]. Data on single LT indications and LT modality (single/bilateral/heart-lung) are not available.

We considered also the number of LTs and age at the surgical procedure recorded by the ICFR in Italy during 2010–2014, to compare these data with those of the CF subjects included in our study [11, 13]. The ICFR did not collect clinical data of CF individuals at time of inclusion on the WL, during the waiting time, at the time of transplantation, during the perioperative period or in the follow-up after the LT.

The study protocol was approved by the Ethics Committee of the Hospitals involved in the survey.

Descriptive statistics and comparisons between groups for continuous variables were performed according to normal distribution tests. Kruskal-Wallis analysis of variance was used to compare variables among three groups of individuals according to their age. The Mann-Whitney test was used to compare demographic data and outcomes of subjects listed in the standard versus the urgent LT programs.

Differences in proportions of categorical data were assessed using the Chi-square test. Statistical significance was defined as *p* < 0.05.

## Results

16/22 (72.7%) CF Centers sent data on CF individuals included on the WL during the 5-year study period. The survey covered 82.4% of subjects included in the 2010 ICFR Report [11].

Two hundred fifty-nine subjects were included on the WL: the median (interquartile range) age was 30.4 (22.1–37.5) years (Table 1). Three subjects were removed from the WL because of improved clinical condition after 2.1, 0.8 and 0.8 years, respectively. Two subjects, retransplanted during the study period, were considered only for their first LT. We recorded 50 deaths (19.3%) during patients’ time on the WL, 159 lung transplants (61.4%) and 47 (18.1%) subjects still on the WL at December 31, 2015 (Table 1).

**Table 1:** Characteristics and outcomes of 259 subjects with cystic fibrosis included on the waiting list for lung transplantation during 2010-2014^a^

	All	Age (yrs)			
		< 17.9	18–29.9	≥ 30	
No.	259	38^c^	88	133^d^	
Age at inclusion on WL (yrs)^b^	30.4 (22.1–37.5)	15.3 (12.5–17.0)	24.4 (21.8–26.1)	37.0 (33.7–42.0)	
Females: No. (%)	148 (57.1)	27 (71.1)	49 (55.7)	72 (54.1)	*p* = 0.061
Deaths on WL: No. (%)	50 (19.3)	9 (23.7)	14 (15.9)	27 (20.3)	*p* = 0.548
Waiting time of deceased subjects (yrs)^b^	0.8 (0.2–1.2)	0.9 (0.2–1.9)	0.5 (0.1–1.2)	0.8 (0.4–1.3)	*p* = 0.639
LTs: No. (%)	159 (61.4)	24 (63.2)	55 (62.5)	80 (60.2)	*p* = 0.913
Waiting time of transplanted sub-jects (yrs)^b^	0.9 (0.3–1.8)	0.8 (0.5–1.3)	0.8 (0.2–1.9)	1.0 (0.4–1.9)	*p* = 0.514
Still on WL: No. (%)	47 (18.1)	3 (7.9)	19 (21.6)	25 (18.8)	*p* = 0.180

*WL* waiting list, *LT* lung transplantation

^a^outcomes were considered until December 31, 2015

^b^data are reported as median and interquartile range

^c^two subjects were removed from the WL after 2.1 and 0.8 years

^d^one subject was removed from the WL after 0.8 years

CF individuals were grouped by age into three groups at the time of inclusion on the WL, with the aim of comparing outcomes between pediatric patients, young adults and subjects older than 30 years. Table 1 shows that more females were recorded among pediatric subjects compared to adults either younger or older than 30 years, but the difference was not statistically significant (*p* = 0.061). There were no statistically significant differences in number of deaths, LTs, subjects still on the WL, and duration of WL between deceased and transplanted subjects in the three groups. The waiting time of deceased subjects on the WL was not different from that of transplanted subjects (Table 1).

Table 2 compares demographic data and outcomes of 57 individuals listed in the IULTp to 202 patients listed only in the standard LT program. There were no differences in sex, age at inclusion on the WL and mortality rate during WL between the two groups. The percentage of subjects who waited less than 3 months for surgery was significantly different in the two groups (43.9% vs 3.5%: *p* < 0.001). Deaths within 3 and 12 months were more frequent in subjects who required an urgent procedure compared to those undergoing standard LT (19.3 and 24.6% vs 6.4 and 8.9%, *p* < 0.01 and *p* < 0.01, respectively).

**Table 2:** Comparison of demography and outcomes between subjects listed for urgent lung transplantation program and those listed for standard lung transplantation program^a^

	Urgent lung transplantation program	Standard lung transplantation program	
No.	57	202^c^	
Females: No. (%)	34 (59.6)	114 (56.4)	*p* = 0.665
Age at inclusion on WL (yrs)^b^	27.6 (23.4–36.7)	30.5 (21.8–37.6)	*p* = 0.826
Deaths on WL: No. (%)	11 (19.3)	39 (19.3)	*p* = 0.998
Waiting time of deceased subjects (yrs)^b^	0.4 (0.2–0.9)	0.8 (0.3–1.4)	*p* = 0.127
LTs: No. (%)	46 (80.7)	113 (55.9)	*p* < 0.001
Waiting time of transplanted subjects (yrs)^b^	0.2 (0.1–1.0)	1.0 (0.6–1.9)	*p* < 0.001
Subjects waiting less than 3 months before LT: No. (%)	25 (43.9)	7 (3.5)	*p* < 0.001
Transplanted subjects deceased during the first 3 months after LT: No. (%)	11 (19.3)	13 (6.4)	*p* < 0.01
Transplanted subjects deceased during the first 12 months after LT: No. (%)	14 (24.6)	18 (8.9)	*p* < 0.01

*WL* waiting list, *LT* lung transplantation

^a^outcomes were considered until December 31, 2015

^b^data are reported as median and interquartile range

^c^three subjects were removed from the WL after 2.1, 0.8 and 0.8 years

The National Transplant Center reported 107 LTs in 2010, 120 in 2011, 114 in 2012, 141 in 2013 and 126 in 2014, including single, bilateral and heart-lung LTs. The mean mortality of those patients on the WL was 11.5% in 2010, 10.2% in 2011, 12.1% in 2012, 10.9% in 2013 and 9.3% in 2014 for all indications for LT [12].

The ICFR reported that there were 20 LTs in CF patients in 2010, 36 in 2011, 25 in 2012, 40 in 2013 and 34 in 2014 [11, 13]. According to the Reports of the National Transplant Center, the primary disease of 155/608 transplant recipients was cystic fibrosis (25.5%) [11–13]. Data on mortality of patients on the WL, number of either urgent or standard LTs, data on mortality after LT were not reported by the ICFR [11–13].

## Discussion

The major findings of our study are the 19.3% mortality rate of those CF patients on the WL and the less favorable outcome soon after LT of those patients who underwent urgent LT, when compared with those undergoing standard procedure.

The introduction of LAS in the USA has helped to reduce the mortality rate for CF patients on the WL from 22 to 29 to 12.9% and benefited survival after LT in recipients with CF [5, 6, 14–16]. Preliminary reports have recorded a decrease in mortality of CF patients on WL from 22.1 to 13.7%, comparing 1-year before and after introduction of LAS in Germany in 2011 [17].

Both dedicated emergency programs in France and Italy were associated with a decrease in mortality of patients on the WL for all indications: in France from 8.6% in 2006 to 3.5% in 2011, in Italy from a range of 11.5–14.7% during 2005–2009 to a range of 9.3–12.1% during 2010–2014 [8–10, 12]. Unfortunately, data about outcomes of different underlying lung diseases is not available in Italy. The mortality rate of CF subjects on the WL in our study remained high, being nearly double that recorded for all indications for LT, including CF, in the same time period, during which the urgent procedure was implemented [15]. The findings on mortality rate in CF may suggest that a different organ allocation policy should be considered in Italy to improve survival for subjects with cystic fibrosis who are on the WL and for those who receive lung transplantation.

A late inclusion in WL and therefore a poor selection of CF patients should be unlikely for the standard LT, since we found that the waiting time was similar in deceased and transplanted individuals. Inclusion and exclusion criteria on the WL were not different from those reported in the literature [1, 2]. However, we found that the waiting time of subjects undergoing urgent LT was significantly less compared to those undergoing standard LT. A waiting time of less than 3 months was recorded in 43.9% of subjects who were moved from standard to urgent LT. An unpredictable, rapid decline in lung function can occur in some patients with CF and moving from standard to urgent LT represents the only opportunity for a lung transplant in these patients.

Donor shortage and the inadequate number of LTs are also reasons for mortality rates in CF patients waiting for a transplant. The ratio between number of LTs and number of subjects on the active WL was 312/490 (0.64) in France, 327/581 (0.56) in Germany and 120/561 (0.21) in Italy in 2011: these figures were associated with different WL durations [8–10, 12, 17, 18].

For patients who moved from standard to urgent LT, IULTp resulted in LT in 80.7% of the cases and should be considered an efficient strategy, considering that mortality while on the WL (19.3%) was the same as that for patients in the standard program (19.3%). The IULTp therefore offered an opportunity for LT to seriously ill CF patients who would have otherwise died while on the WL. However, the IULTp was associated with increased mortality within the first year after LT. The urgent LT program in France showed similar results: the overall in-hospital mortality rate was 29.4% for 95 transplanted subjects, with extracorporeal membrane oxygenation (ECMO) prior to LT being the sole independent mortality risk factor [8]. The high mortality associated with IULTp should be interpreted cautiously in the context of the most critical conditions in which it was used. A high LAS score was also associated with an increased risk of death after LT in cystic fibrosis patients [19]. Prospective research is needed to assess whether timing, duration and modality of ECMO and associated invasive respiratory support could be modified to decrease the mortality rate after LT.

The main limitation of our survey is that we included only data on the mortality rate of CF patients on the WL and during the first year after LT. We did not record the clinical characteristics of CF individuals at the time of inclusion on the WL, the deterioration of lung disease during the waiting time, lung donor characteristics, the modality and duration of invasive support for patients in the urgent program before and after LT, all of which are risk factors affecting survival before and after LT [1–5, 8, 14, 15]. Many of these clinical data were recorded by the Transplant Centers, but were not included in our survey. Neither the ICFR nor the National Transplant Center collected or reported this important clinical information. The ICFR recently reported that 10% of transplanted subjects had missing data on outcomes after LT [13]. Probably some patients were followed up only by the Transplant Centers once they received a LT. A prospective study should be designed which involves both CF Centers and Lung Transplant Centers to collect clinical data for assessing which risk factors affect survival before and after LT.

Our survey covered 82.4% of CF subjects in Italy during our study period and our mortality rates could be underestimated. The ICFR recently reported that 155 CF patients received a LT in the same 5-year period [11, 13]. Our study reported 159 LTs for 259 subjects included on the WL. Although a bias of missing data cannot be excluded, our study recorded mortality data of all 259 subjects included on the WL between 2010 and 2014.

## Conclusions

In conclusion, despite some limitations, this study is the first attempt to evaluate mortality of Italian CF patients while they are on the waiting list for lung transplantation and within 3 and 12 months after they have received either urgent or standard LTs. In spite of the introduction of emergency LT in Italy, the mortality rate of CF patients on the WL was twice that for all LT indications, including CF, and higher than that recorded in other countries for the same disease. The urgent procedure was associated with a less favorable outcome soon after LT.

To decrease the mortality of CF patients on the WL and to improve outcomes after LT, health authorities need to acknowledge different aspects of the LT program, from the shortage of suitable lung donors to organ allocation policies. The use of marginal grafts, the reconditioning of grafts with poor function and from donation after cardiac death and living lobar transplant have been developed to increase the number of LTs. The current organ allocation policy in Italy should also be critically evaluated in relation to the results obtained in CF subjects: disease-specific parameters should be considered for waitlist death risk and transplant benefit to give priority to LT. A national registry that is linked with the transplant centers and which collects demographic and clinical data of all subjects at the time of their inclusion on the waiting list, as well as outcome after LT, is a top priority for improving the organ allocation policy for all LT indications in Italy.

(Manuscript: “Mortality rate of patients with cystic fibrosis on the waiting list and within one year after lung transplantation: a survey of Italian CF Centers”)
